# Changes in physiotherapy students’ beliefs and attitudes about low back pain through pre-registration training

**DOI:** 10.1186/s40945-021-00106-1

**Published:** 2021-05-17

**Authors:** Guillaume Christe, Ben Darlow, Claude Pichonnaz

**Affiliations:** 1 Department of Physiotherapy, HESAV School of Health Sciences, HES-SO University of Applied Sciences and Arts Western Switzerland, Lausanne, Switzerland; 2 Swiss BioMotion Lab, Department of Musculoskeletal Medicine, University Hospital and University of Lausanne (CHUV-UNIL), Lausanne, Switzerland; 3 Department of Primary Health Care and General Practice, University of Otago, Wellington, New Zealand; 4 Department of Musculoskeletal Medicine, University Hospital and University of Lausanne (CHUV-UNIL), Lausanne, Switzerland

**Keywords:** Psychological factors, Low back pain, Education, Biopsychosocial

## Abstract

**Background:**

Implementation of best-practice care for patients with low back pain (LBP) is an important issue. Physiotherapists’ who hold unhelpful beliefs are less likely to adhere to guidelines and may negatively influence their patients’ beliefs. Pre-registration education is critical in moving towards a biopsychosocial model of care. This study aimed to investigate the changes in 2nd year physiotherapy students’ beliefs about LBP after a module on spinal pain management and determine whether these changes were maintained at the end of academic training.

**Methods:**

During three consecutive calendar years, this longitudinal cohort study assessed physiotherapy students’ beliefs with the Back Pain Attitudes Questionnaires (Back-PAQ) in their 1st year, before and after their 2nd year spinal management learning module, and at the end of academic training (3rd year). Unpaired t-tests were conducted to explore changes in Back-PAQ score.

**Results:**

The mean response rate after the spinal management module was 90% (128/143 students). The mean (± SD) Back-PAQ score was 87.73 (± 14.21) before and 60.79 (± 11.44) after the module, representing a mean difference of − 26.95 (95%CI − 30.09 to − 23.80, *p* < 0.001). Beliefs were further improved at the end of 3rd year (− 7.16, 95%CI − 10.50 to − 3.81, *p* < 0.001).

**Conclusions:**

A spinal management learning module considerably improved physiotherapy students’ beliefs about back pain. Specifically, unhelpful beliefs about the back being vulnerable and in need of protection were substantially decreased after the module. Improvements were maintained at the end of academic training one-year later. Future research should investigate whether modifying students’ beliefs leads to improved clinical practice in their first years of practice.

## Background

Low back pain (LBP) is the leading cause of disability worldwide and is associated with significant reduction in quality of life and severe economic burden [1, 2]. Unhelpful attitudes and beliefs about back pain have been shown to be predictors of outcomes [3]. People commonly believe that the back is vulnerable to injury and needs protection [4–8] and these beliefs may contribute to pain-related fear, catastrophizing and anxiety [9–12]. These psychological factors are important predictors of unhelpful behaviours and elevated levels of disability [13–16].

Gaps between evidence and practice in the management of LBP have been identified worldwide indicating that many patients receive sub-optimal care [17, 18]. While there are many factors that influence implementation of best-practice care, evidence suggests that unhelpful beliefs among health professionals is a significant factor associated with reduced guideline adherence [19–22]. Therefore, addressing health professionals’ unhelpful beliefs has been strongly recommended to improve the quality of care of LBP [11, 17, 18].

Physiotherapists are at the frontline of LBP management and spend a considerable amount of time with patients [18, 23]. Consequently, physiotherapists have the opportunity to significantly influence patients’ beliefs and behaviours (positively or negatively) and, in turn, influence recovery outcomes [11, 12, 19, 22]. Physiotherapists’ beliefs can also strongly influence their clinical decisions and delivery of core guideline recommended treatments [22, 24], such as movement, physical activity and self-management.

While the biopsychosocial model of LBP is largely recognized, management of patients with LBP within a predominantly biomedical framework is still very frequent among physiotherapists [17, 18, 22, 24]. It has been argued that the focus of entry-level education on anatomical, pathological and physical dysfunctions contribute to this problem and hinder the transition towards a biopsychosocial model of care [25], while teaching about the multidimensional nature of LBP and current evidence is an important step toward implementation of the biopsychosocial model in future practice and, ultimately, improve care for patients with LBP [17, 18].

Unhelpful beliefs are prevalent amongst physiotherapy students, albeit to a lesser extent than other health care professions, but highly variable depending of the country and the stage of training [26–31]. There is limited information on training approaches that are effective in improving students’ beliefs. Two studies found positive changes in physiotherapy students’ beliefs following biopsychosocially-orientated LBP learning [29, 32]. These studies assessed either students’ beliefs about whether pain justified activity limitation and disability or that back pain is likely to have negative future consequences. However, longitudinal changes in physiotherapy students’ beliefs about their own back or their attitudes about movement, activity, and recovery behaviours or the impact of specific learning on these has not been investigated.

The main objective of this study was to investigate changes in 2nd year physiotherapy students’ attitudes and beliefs about LBP following completion of a biopsychosocially informed spinal pain management learning module. The secondary objective was to determine whether any changes following the module were maintained at the end of academic education (3rd year). We hypothesized that helpful attitudes and beliefs would be more prevalent among physiotherapy students after completing the spinal pain management module (2nd year) and that these changes would be maintained at the end of academic education.

## Methods

### Study design

This study is a longitudinal observational cohort study and was written according to the Strengthening the Reporting of Observational Studies in Epidemiology (STROBE) criteria [33].

### Participants

**Fig. 1:**
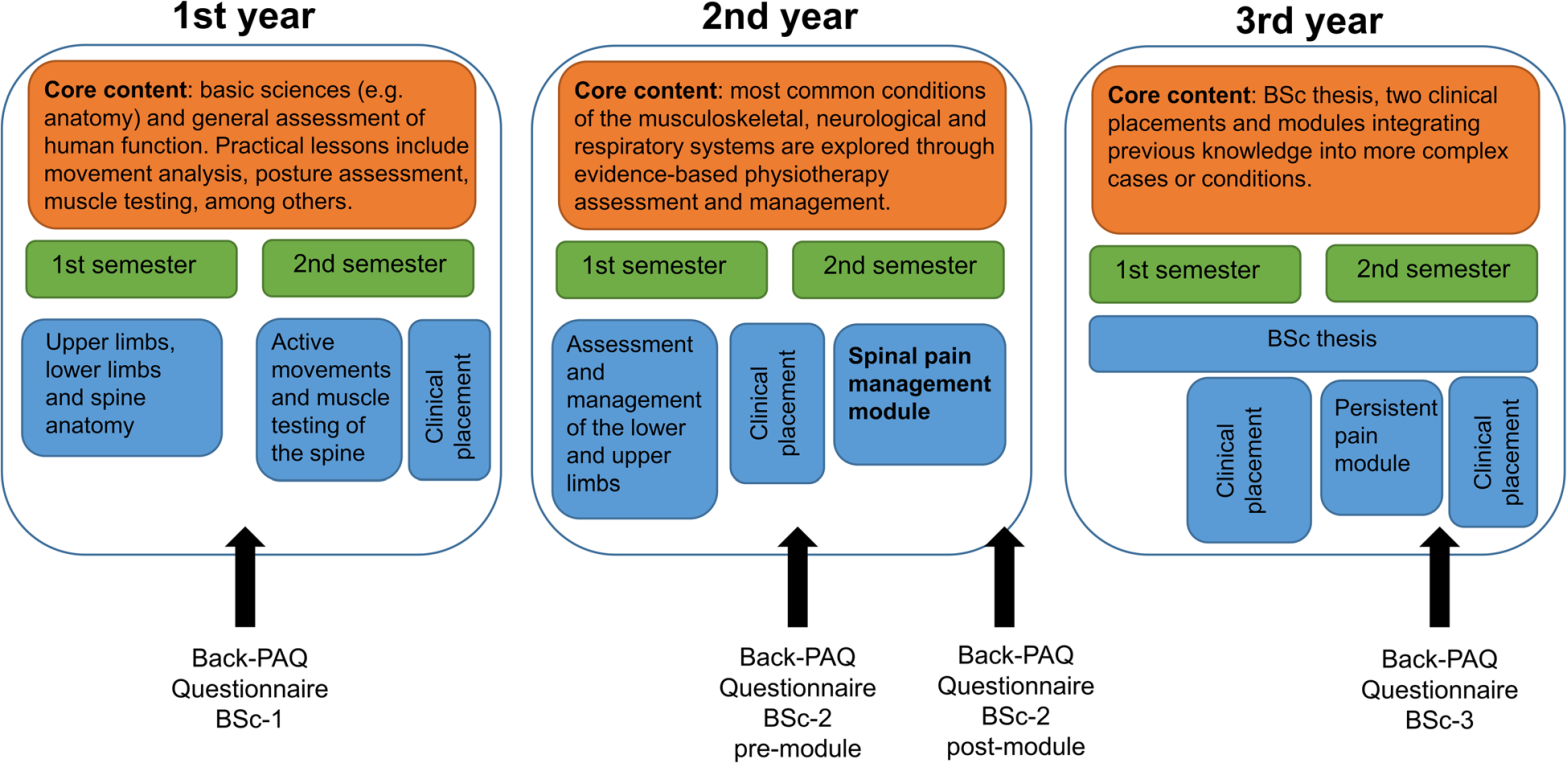
Assessment of attitudes and beliefs during the physiotherapy program. Only information relevant to this study are included in the figure. BSc-1: first year students; BSc-2: second year students; BSc-3: third year students

During three consecutive years (2018 to 2020), three cohorts of pre-registration physiotherapy students at Haute Ecole Santé Vaud (HESAV) School of Health Sciences (Lausanne, Switzerland) were invited to participate anonymously in the study. Students received an email invitation for a Google Forms questionnaire at the beginning of the second semester (first year students – BSc-1), before and immediately after a spinal pain management learning module (second year students – Bsc-2) and at the end of the last mandatory module of the pre-registration training (third year students – BSc-3) (Fig. 1). Because the study was conducted from 2018 to 2020, only one cohort (2018–2020) had data collected at all timepoints. No BSc-1 data were collected for the 2017–2019 cohort and no BSc-3 data were collected for the 2019–2021 cohort. The local Research Ethics Committee (CER-VD) confirmed that the project complied with Swiss ethical regulations on studies without identifying data collection (REQ-2018-00146). Participants received information about the study and the right not to participate, and gave their informed consent before completing the questionnaire.

### Physiotherapy program

The physiotherapy course at HESAV is a three-year pre-registration Bachelor of Science (BSc) program of 180 European Credits Transfer System (ECTS). Musculoskeletal content of each academic year is briefly described in Fig. 1. The spinal pain management learning module, taught in the second year of the program, is a 6-ECTS module, that covers assessment and management of pelvic, lumbar, thoracic and cervical pain conditions. Within this module, students had 9 lectures (90-min each) about differential diagnosis, current understanding of LBP, and recommendations for assessment and management of non-specific and specific LBP delivered by medical doctors (5 lectures) and academic physiotherapists (4 lectures). In addition, there were 6 practical lessons (3 h each) that covered manual assessment and treatment of clinical cases (2 lessons), progressive and functional exercises (2 lessons), and management of low back-related leg pain (2 lessons). Finally, a three-hours training activity with a focus on communication skills and individual exercise prescription was conducted with simulated patients. Important foci of the module were developing a biopsychosocial understanding of LBP and discussing common misconceptions about LBP (Table 1). Furthermore, students were encouraged to move towards a positive health concept that emphasised the capacity of individuals to adapt and self-manage [17]. The module encouraged progressive loading in daily-life activities to increase tolerance and decrease sensitivity to pain, rather than protecting the back to decrease symptoms (as would be advocated in a traditional biomedical approach). The final academic module of third year students focussed on management of long-term conditions (5-ECTS), particularly persistent pain. In this module, students had two lectures on pain mechanisms (90 min each) and multiple activities based on complex clinical cases to foster a biopsychosocial understanding of chronic pain. They also had a learning activity with simulated patients to foster communication skills (especially building a shared understanding). The BSc-2 spinal pain management module was delivered exclusively online in 2020 due to the COVID-19 pandemic. Online learning included asynchronous lectures and group activities, in which students had to answer questions about their understanding of spinal conditions, demonstrate video-based exercises and propose optimal assessment and management strategies for various patients’ situations based on clinical vignettes. They did not have any practical manual therapy learning. The training activities with simulated patients were also cancelled.

**Table 1:** Concepts targeted during the spinal management module in BSc-2

Unhelpful beliefs	Messages delivered during the BSc-2 module
Back pain is due to structural damage	• Degenerative changes are frequent in asymptomatic population [2, 34] • Little association between degenerative changes and the level of pain and disability [35, 36]
LBP is a serious condition	• LBP is very frequent and normal [2, 37] • LBP due to serious pathology is rare [2, 38, 39]
Biomedical or biomechanical factors are the major cause of LBP	• LBP is a multidimensional condition [2, 40]
It is necessary to find the source of pain to treat LBP	• It is difficult/impossible to accurately determine the tissue source of LBP [2] • Identifying the source of pain does not lead to better outcomes [2, 41]
LBP is due to “something” out of place that needs to be corrected	• LBP is not due to “something” out of place [2, 42, 43] • Manual therapy has short term effect and works as a pain modulating technique (no structural changes following manual therapy) [18, 44] • Guidelines recommend active exercises as first line treatment [18, 45]
Bending/lifting with round back is dangerous for the back	• Biomechanical studies do not consistently support that lifting with a straight back is better [46, 47] • Epidemiological studies do not support flexion as an independent risk factor for LBP disability [48] • Manual handling training (doing less flexion) has no effect on LBP [51, 52] • Patients with LBP move with a more rigid spine (less flexion and more muscle activity) [53–55] • Psychological factors are associated with a more rigid movement [16]
There is right and wrong ways to move	• Movement is very variable and there is no right or wrong way to move [54, 58, 59] • Confidence to move seems more important than how you move [13] • If a movement is painful, you can temporarily adapt it. But in the long term, all movements should be promoted and trained (improving tolerance) [60]
The back is vulnerable and needs to be protected	• Loading has positive effects on the back [61, 62] • Disuse has negative effects on the back [63] • The back can positively adapt to load [64]
Bad postures (particularly slumped postures) cause back pain	• There is no right or wrong posture [65, 66] • Posture is very variable [67] • Lumbar spine posture is not an independent risk factor for LBP [68] • Patients with LBP often show a hyperactivity of trunk muscles [55, 69]
Core stabilisation exercises are important to treat LBP	• Patients with LBP move with a more rigid spine (and naturally adopt more “neutral” postures) [53] • There is no association between transversus abdominus or lumbar multifidus activation and clinical outcomes [70, 71] • Stabilisation exercises are not more effective than other types of exercises [72, 73] • The idea that the back needs to be stabilized may elevate fear avoidance beliefs [11, 72]
Important factors that need to be modified during physiotherapy treatment are muscle strength and mobility (physical factors)	• Improvement in physical factors alone do not explain improvement in disability [74] • Self-efficacy, pain-related fear and psychological distress are important to address [75, 76] • Physiotherapy intervention can improve psychological factors through education and active treatment (e.g. gradual exposure, promoting self-efficacy) [78]

*LBP* Low back pain

### Outcomes

The primary outcome was the validated French version of the Back Pain Attitudes Questionnaire (Back-PAQ) [5, 80]. The questionnaire is composed of 34 items scoring from 1 to 5 points on a Likert scale (False, Possibly false, Unsure, Possibly true, True). Higher total score (range 34 to 170) indicates more unhelpful beliefs and attitudes about LBP. The questionnaire items and themes were created based on findings from qualitative studies with people with LBP [4, 5]. The six different themes are ‘the vulnerability of the back’ (vulnerability), ‘the need to protect the back’ (protection), ‘the correlation between pain and injury’ (pain), ‘the special nature of back pain’ (special pain), ‘activity participation while experiencing back pain’ (activity) and ‘the prognosis of back pain’ (prognosis). Students also gave details about their age and gender.

### Statistical analysis

The mean Back-PAQ total score was calculated for each study time (BSc-1, BSc-2 pre module, BSc-2 post module and BSc-3). Unpaired t-tests were conducted to determine whether there were differences in Back-PAQ total score before and after the module for the three cohorts together and for each cohort separately. Because students completed the questionnaire anonymously, paired t-test could not be used. When possible, unpaired t-tests were conducted to test differences in Back-PAQ score between the end of the module and the end of pre-registration academic training as well as between BSc-1 and BSc-2 pre-module. Mean scores and mean differences per Back-PAQ item were also calculated before and after the spinal pain management module. There were no missing data in the questionnaires (all answers were compulsory to submit the questionnaire). Statistical analyses were performed with SPSS (Version 23, IBM, NY, USA), using a significance level corrected for the eight statistical tests and set a priori at α < 0.006.

## Results

The response rate and number of students that participated in the study was 90% (95 students) in BSc-1, 92% (132 students) in BSc-2 before the module, 90% (128 students) in BSc-2 after the module and 87% (80 students) in BSc-3. Their mean age (SD) was 23.8 (2.9) years and 68.3% were female. Participant characteristics, response rates and mean Back-PAQ score at each time point for each cohort are presented in Table 2. Mean Back-PAQ scores reduced following the spinal pain management learning module in the 2017–2019 (− 27.36, 95%CI − 33.04 to − 21.68, *p* < 0.001), 2018–2020 (− 21.91, 95%CI − 26.84 to − 16.98, *p* < 0.001) and 2019–2021 (− 31.49, 95%CI − 36.21 to − 26.77, *p* < 0.001) cohorts. The pooled mean Back-PAQ change across cohorts following module completion was − 26.95 (95%CI − 30.09 to − 23.80, *p* < 0.001).

**Table 2:** Characteristics and Back-PAQ scores at each study time point

Cohort	Study time	Age (mean)	Female (%)	N	Response rate (%)	Back-PAQ score	95%CI
2017–2019	BSc-2 pre module (2018)	23.4	68.4	38/45	84	95.6	[91.7 to 99.4]
	BSc-2 post module (2018)	23.5	75.7	37/45	82	68.2	[64.3 to 72.1]
	BSc-3 (2019)	24.8	66.7	39/43	91	60.9	[57.1 to 64.7]
2018–2020	BSc-1 (2018)	22.4	62	50/52	96	94.8	[91.4 to 98.1]
	BSc-2 pre module (2019)	23.3	68.1	47/50	94	82.7	[79.2 to 86.1]
	BSc-2 post module (2019)	23.4	63.6	44/50	88	60.8	[57.2 to 64.3]
	BSc-3 (2020)	24.8	65.9	41/49	84	53.3	[49.6 to 57]
2019–2021	BSc-1 (2019)	23.3	71.1	45/53	85	95.8	[92.3 to 99.4]
	BSc-2 pre module (2020)	24.1	70.2	47/48	98	86.5	[83 to 89.9]
	BSc-2 post module (2020)	24.3	78.7	47/48	98	55	[51.5 to 58.4]

N: number of participants included in the study (first number) in relation to the total number of students in this cohort (second number)

**Fig. 2:**
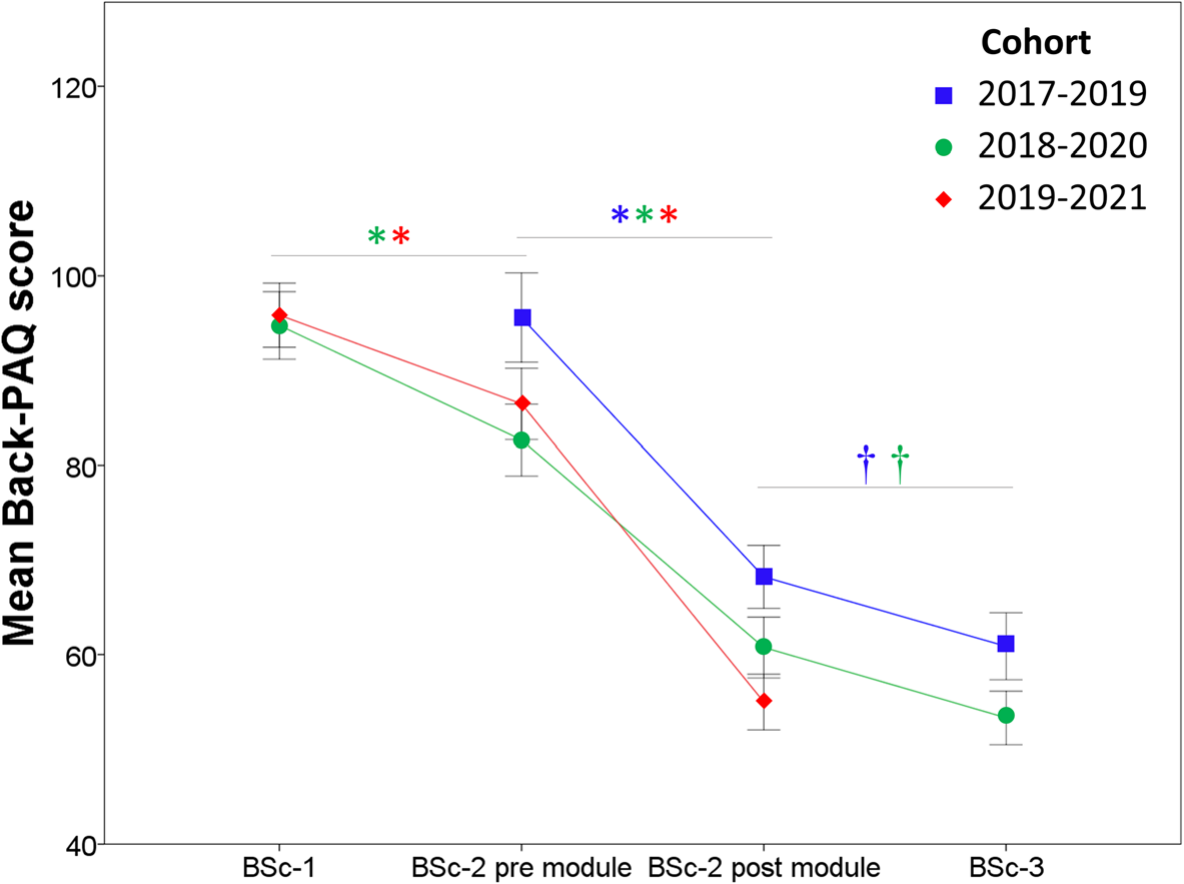
Back-PAQ scores at each study time point for the three cohorts with longitudinal data. Cohorts are named based on their start and end year of study (e.g 2019–2021 cohort is equivalent to 2019 BSc-1 and 2020 Bsc-2). *: *p* < 0.001; †:*p* < 0.005 (colours are related to the corresponding cohort)

The Back-PAQ score further reduced between the end of the module and the end of BSc-3 for both the 2017–2019 (− 7.34, 95%CI − 12.12 to − 2.57, *p* = 0.003) and the 2018–2020 (− 7.43, 95%CI − 11.67 to − 3.19, *p* = 0.001) cohorts (data not available for the 2019–2021 cohort). The pooled mean Back-PAQ change of these two cohorts was − 7.16 (95%CI − 10.50 to − 3.81, *p* < 0.001). Mean differences between BSc-1 and BSc-2 pre module were also statistically significant for the 2018–2020 (− 12.10, 95%CI − 17.23 to - 6.98, *p* < 0.001) and the 2019–2021 (− 9.38, 95%CI − 14.39 to − 4.36, *p* < 0.001) cohorts (Fig. 2). Pooled mean Back-PAQ change was − 10.71 (95%CI − 14.28 to − 7.14, *p* < 0.001). Mean score per item and mean differences before and after the module are presented in Table 3.

**Table 3:** Back-PAQ items score before and after the spinal pain management module

Question	Bsc-2 pre-module		Bsc-2 post-module		Mean difference	95%CI
	Mean	SD	Mean	SD		
8) Good posture is important to protect your back	4.00	1.17	1.95	1.30	2.05	1.74 – 2.35
5) Lifting without bending the knees is not safe for your back	3.08	1.59	1.20	0.72	1.88	1.58 – 2.18
11) You could injure your back if you are not careful	3.62	1.22	1.74	1.04	1.88	1.60 – 2.16
6) It is easy to injure your back	3.10	1.46	1.53	0.93	1.57	1.27 – 1.87
22) If you ignore back pain, you may cause damage to your back	3.55	1.15	2.04	1.32	1.51	1.20 – 1.81
9) If you overuse your back, it will wear out	2.85	1.29	1.65	1.05	1.20	0.91 – 1.49
^a^28) Most back pain settles quickly, at least enough to get on with normal activities	2.67	1.18	1.48	0.89	1.19	0.94 – 1.44
24) To effectively treat back pain you need to know exactly what is wrong	3.14	1.37	2.01	1.44	1.13	0.79 – 1.47
^a^29) Worrying about your back can delay recovery from back pain	2.31	1.05	1.28	0.61	1.03	0.82 – 1.24
^a^1) Your back is one of the strongest parts of your body	2.13	1.24	1.13	0.35	1.00	0.78 – 1.23
^a^3) Bending your back is good for it	2.07	1.26	1.13	0.36	0.94	0.71 – 1.16
33) There is a high chance that an episode of back pain will not resolve	2.58	1.22	1.67	1.01	0.90	0.63 – 1.18
14) A twinge in your back can be the first sign of a serious injury	2.57	1.22	1.76	1.17	0.81	0.52 – 1.10
7) It is important to have strong muscles to support your back	4.37	1.01	3.56	1.45	0.81	0.50 – 1.12
23) It is important to see a health professional when you have back pain	3.99	0.98	3.19	1.40	0.80	0.51 – 1.10
^a^30) Focussing on things other than your back helps you to recover from back pain	2.36	0.98	1.60	0.83	0.76	0.54 – 0.98
12) You can injure your back and only become aware of the injury sometime later	4.22	0.99	3.48	1.43	0.74	0.44 – 1.04
4) Sitting is bad for your back	2.42	1.20	1.69	1.14	0.73	0.44 – 1.02
10) If an activity or movement causes back pain, you should avoid it in the future	2.08	1.03	1.40	0.89	0.68	0.45 – 0.92
^a^2) Your back is well designed for the way you use it in daily life	1.71	0.96	1.08	0.37	0.63	0.46 – 0.81
^a^31) Expecting your back pain to get better helps you to recover from back pain	2.23	1.05	1.70	1.15	0.53	0.26 – 0.80
^a^17) When you have back pain, you can do things which increase your pain without harming the back	1.90	0.99	1.41	0.88	0.49	0.26 – 0.72
32) Once you have had back pain there is always a weakness	1.71	0.89	1.23	0.70	0.48	0.28 – 0.67
26) When you have back pain the risks of vigorous exercise outweigh the benefits	2.15	1.10	1.68	1.07	0.47	0.21 – 0.74
19) It is worse to have pain in your back than your arms or legs	3.24	1.25	2.81	1.47	0.43	0.10 – 0.76
20) It is hard to understand what back pain is like if you have never had it yourself	3.98	1.04	3.55	1.39	0.42	0.12 – 0.72
13) Back pain means that you have injured your back	1.58	0.91	1.19	0.60	0.40	0.21 – 0.58
18) Having back pain makes it difficult to enjoy life	4.14	1.00	3.75	1.25	0.39	0.11 – 0.66
^a^15) Thoughts and feelings can influence the intensity of back pain	1.28	0.50	1.03	0.17	0.25	0.16 – 0.34
34) Once you have a back problem, there is not a lot you can do about it	1.26	0.57	1.03	0.22	0.23	0.12 – 0.33
^a^16) Stress in your life (financial, work, relationship) can make back pain worse	1.26	0.52	1.07	0.26	0.19	0.09 – 0.29
^a^27) If you have back pain you should try to stay active	1.19	0.48	1.02	0.12	0.17	0.09 – 0.26
25) If you have back pain you should avoid exercise	1.24	0.58	1.09	0.31	0.16	0.04 – 0.27
21) If your back hurts, you should take it easy until the pain goes away	1.76	0.94	1.66	1.19	0.10	−0.16 – 0.36

The items are ordered from the largest change during the module to smallest change. Lowest scores at associated with more helpful beliefs (1 = false and 5 = true). ^a^ scores are reversed for items worded in the reverse direction so that a lower score also indicates that the helpful belief is more strongly held

## Discussion

Physiotherapy students had predominantly unhelpful beliefs about back pain when they entered the course and these beliefs improved during each year of their training. Second year physiotherapy students’ beliefs became considerably more helpful after completing a learning module that aimed to communicate recent evidence and develop a biopsychosocial understanding of LBP.

While previous cross-sectional studies already demonstrated differences in students’ beliefs between different academic years [26, 30, 31], with more experienced students having more positive beliefs, our results showed that the largest change occurred right after a spinal pain management module, while smaller changes occur before and after this topic was specifically addressed. These changes were consistent and large for the three cohorts and were all above the minimal detectable change (MDC) of the Back-PAQ (14.5 points) [80]. Conversely, changes before and after the module were below the MDC. These results suggest that a biopsychosocially-orientated learning module with a targeted pedagogical approach can effectively improve back pain beliefs among future health professionals. Educators and pre-registration programs should consider integrating similar modules to foster their helpful beliefs that are associated with guideline concordant practice.

The large changes in beliefs about LBP that occurred as a result of the spinal pain management learning module may have resulted from several factors. First, the current multidimensional understanding of LBP and evidenced-based management strategies were frequently discussed to highlight the importance of active strategies and self-care management. Moreover, the ideas that the back can positively adapt to load and that protection does not offer long-term positive effects were central. These concepts were integrated during practical sessions covering exercise progression and the activity with simulated patients. This module used an active learning strategy to foster reflection and discuss disruptive concepts for students. As an example, how and why lumbar flexion can be progressively included in progressive loading exercises was frequently discussed with students as beliefs about the danger with loaded flexion were very prevalent before the module. This module used an integrative approach of both scientific evidence and practical courses to foster a positive image of the back and hinder prevalent unhelpful messages about ergonomic, protection and vulnerability. This consistent message throughout the module may have positively influenced students’ beliefs.

Previous studies have demonstrated that students’ beliefs about the relationship between LBP and physical function can be improved with specific training [29, 32]. Our findings extend these results by demonstrating positive changes in students’ beliefs about their own back and how they should respond to back pain and that these changes were maintained one-year later. Importantly, while changes in beliefs occurred in all items of the Back-PAQ, the questions with the largest changes were mostly related to the beliefs that the back is easy to injure (eg, questions 1, 5, 6, 9, 22 about vulnerability) and needs protection (eg, questions 8, 11). These changes are notable as physiotherapists who hold these beliefs have been found to make less evidenced-based clinical decisions and provide more advice that movement should be avoided [24]. Thus, following the spinal pain management training module, students may be more prepared to deliver adequate messages concerning these unhelpful beliefs, which are very prevalent in people with and without LBP [4–8] and have been associated with important contributors to LBP disability, such as pain-related fear, catastrophizing and anxiety [9–12].

The COVID-19 pandemic required rapid adaption of the 2020 physiotherapy programmes and a transition to exclusively online learning. For this cohort, the home-based practical courses were exclusively dedicated to exercise progressions and no manual therapy was practiced. The improvement in Back-PAQ score that occurred in this online-only cohort was larger than that of the two previous cohorts with a face-to-face module. While our design precludes any comparison between online or face-to-face modules, this suggests that an online module using active learning strategies is also an effective mechanism to improve physiotherapy students’ beliefs about back pain.

Unhelpful beliefs were relatively prevalent in first year students. These beliefs were more prevalent than amongst practising physiotherapists but less prevalent than in the general population from the same geographic area [8, 24]. These beliefs improved to a small degree over the students’ first year of training (below the questionnaire MDC), suggesting that non-specific education has only a small effect on unhelpful beliefs about LBP and that specific training is needed. Students’ Back-PAQ total and individual item scores following training indicated that their beliefs were more positive than those found in practising physiotherapists in Switzerland [24]. This change may enable these graduates to positively influence the beliefs of their patients and their peers and improve the quality of LBP management.

Future research is necessary to determine whether the changes in LBP beliefs among physiotherapy students are associated with changes in their clinical decisions. Ultimately, it is necessary to understand whether these changes improve the implementation of evidenced-based care in the first years of clinical practice and beyond. Given all the factors that are known to influence guideline implementation [81–84], further intervention may be necessary post-graduation to maintain or further improve beliefs about back pain and integration of evidence-based care. Given the prevalence of unhelpful beliefs in health care professionals and the efficiency of targeted learning demonstrated in these student cohorts, there may also be an opportunity to develop educational strategies for practising physiotherapists. Online learning may be an effective mechanism to deliver this at scale given the positive changes observed in students who learned exclusively online. Qualitative research on perceived efficiency of educational interventions about LBP beliefs may also improve our understanding of physiotherapy students’ learning experience and identify opportunities to refine educational strategies or support ongoing change.

The finding that a targeted active educational program positively modifies beliefs about LBP is likely to be transferable, but the magnitude of change and final level may have also been influenced by the global training environment and module timing in curriculum. The absence of a control group is a key limitation of this study and leaves open the possibility that the changes observed were due to other factors. However, measuring beliefs immediately before and after the spinal learning module reduced the risk that other learning had influenced these changes. It is often not feasible in an educational environment to randomise students to different learning interventions. The design of this study is analogous to a Single Case Experiment Design (SCED). Within SCED, three consistent replications of experimental are considered to increase the internal validity of the study, which was the case in this study for the three before-after specific module significant differences above MDC [85]. Students also completed a BSc-3 module that included content about persistent pain within a biopsychosocial framework, which may have reinforced the messages delivered in the specific LBP management module. This means that we cannot determine whether the spinal pain learning module has a long term effect or whether multiple interventions are required to maintain the positive beliefs developed. We did not record the students’ identification numbers and this precluded the use of statistical analyses based on paired tests such as repeated-measures models. Nevertheless, the unpaired t-tests used in this study demonstrated highly significant changes in beliefs, despite the reduced statistical power of this technique, making a type 2 error unlikely. The high response rate at all time points increases confidence that the findings represent real changes in beliefs across the student cohort, rather than being biased by those with less helpful beliefs selectively dropping out of the study.

## Conclusion

This study found that a biopsychosocially-orientated learning module using active training methods significantly and substantially improved physiotherapy students’ beliefs about LBP. The largest changes occurred in the beliefs that the back is vulnerable and requires protection. Future research is necessary to understand if these changes in beliefs lead to more optimal clinical decisions and enhance high value care for newly graduated.

## Data Availability

The data used in this study are available on request from the corresponding author.
